# Anomalous plantar intrinsic foot muscle attaching to the medial longitudinal arch: possible mechanism for medial nerve entrapment: a case report

**DOI:** 10.1186/s13256-021-02676-x

**Published:** 2021-02-13

**Authors:** R. Claire Aland, Alana C. Sharp

**Affiliations:** 1 School of Rural Medicine, University of New England, Armidale, NSW Australia; 2 School of Biomedical Sciences, University of Queensland, Brisbane, QLD Australia; 3 Institute of Life Course and Medical Sciences, University of Liverpool, Liverpool, UK; 4 School of Science & Technology, University of New England, Armidale, NSW Australia

**Keywords:** Anatomical variation, Medial longitudinal arch, Medial nerve entrapment, Plantar intrinsic muscle, Case report

## Abstract

**Background:**

Muscular variations are potentially symptomatic and may complicate imaging interpretation. Intrinsic foot musculature and extrinsic tendon insertion variations are common. Distinct supernumerary muscles are rare. We report a novel anomalous intrinsic foot muscle on the medial longitudinal arch.

**Case presentation:**

An accessory muscle was encountered on the medial arch of the right foot of a 78-year-old white male cadaver, between layers two and three of the foot intrinsics. It did not appear to be a slip or variant of a known foot muscle. This muscle consisted of two slips that ran transversely on the plantar aspect of the medial arch, crossing the medial transverse tarsal joint and attaching to the tuberosity of the navicular, the short and long plantar ligaments, and spring ligament.

**Conclusions:**

The medial plantar vessels and nerve passed from deep to superficial between the two slips, and this suggests a possible location for medial nerve entrapment.

## Background

The plantar foot contains four layers of intrinsic muscles, interspersed with tendons of extrinsic leg muscles that insert into the foot. The first, most superficial layer contains the flexor digitorum brevis, abductor hallucis, and abductor digiti minimi; the second layer contains the quadratus plantae and four lumbricals; the third contains the adductor hallucis, flexor hallucis brevis, and flexor digiti minimi; the fourth and deepest layer contains the dorsal and plantar interossei.

Several muscles move the hallux, or great toe: in the first layer, the abductor hallucis (AbH) arises from the medial calcaneus, plantar aponeurosis, and flexor retinaculum to insert distally on the medial proximal hallucial phalanx base. In the second layer, the flexor hallucis longus tendon passes deep to the flexor digitorum longus tendon to insert on the base of the distal hallucial phalanx. The third layer contains the two heads of both the adductor hallucis (AdH) and flexor hallucis brevis (FHB). The oblique head of the AdH originates laterally from the cuboid, lateral cuneiform and from the bases of the second and third metatarsals and inserts on the lateral base of the proximal hallucial phalanx, while the transverse head arises from metatarsophalangeal joints of the third–fifth digits. The medial and lateral heads of the FHB originate from the cuneiforms and plantar calcaneonavicular (spring) and long plantar ligaments and insert, via the medial and lateral sesamoids of the first metatarsophalangeal joint, to the proximal hallucial phalanx. In the fourth layer, the tibialis posterior tendon inserts to the navicular and medial cuneiform, and the tibialis anterior tendon inserts to the medial cuneiform and first metatarsal. The medial plantar nerve supplies the AbH and FHB, while the deep branch of the lateral plantar nerve supplies the AdH [16].

Muscular variations are potentially symptomatic and may complicate imaging interpretation. Intrinsic foot musculature and extrinsic tendon insertion variations are common [12–14, 17]. Distinct supernumerary muscles are rare. We report a novel anomalous intrinsic foot muscle on the medial longitudinal arch, which may be a variant of AdH.

## Case presentation

**Fig. 1:**
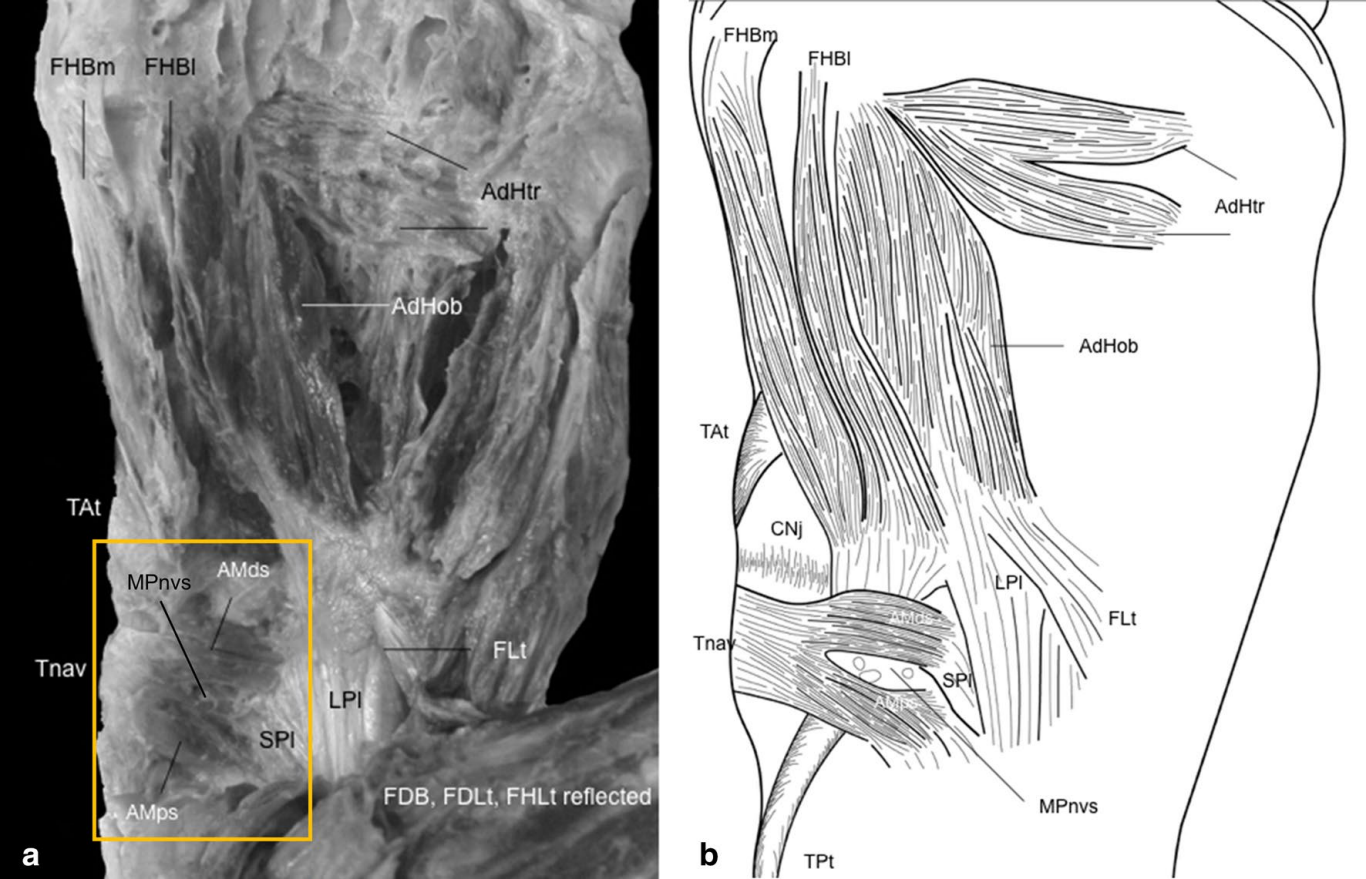
Anomalous muscle slips attach to the medial longitudinal arch: **a** photograph; **b** schematic drawing. Abductor hallucis (AbH) is removed, and the flexor digitorum longus (FDL) and flexor hallucis longus (FHL) tendons and the flexor digitorum brevis (FDB) have been reflected. *AdHob* Adductor hallucis oblique head; *AdHtr* adductor hallucis transverse head; *AMds* anomalous muscle distal slip; *Amps* anomalous muscle proximal slip; *CNj* cuneonavicular joint; *FDB* flexor digitorum brevis *FDLt* flexor digitorum longus tendon; *FHLt reflected* flexor hallucis longus tendon, reflected; *FLt* fibularis longus tendon; *LPl* long plantar ligament; *MPnvs* medial plantar nerves and vessels, *SPl* short plantar ligament, *Tat* tibialis anterior tendon, *Tnav* navicular tuberosity, *TPt* tibialis posterior tendon

This anomalous muscle (Fig. 1) was discovered by dissection at the School of Rural Medicine, University of New England, Australia. The dissection was approved by the UNE Human Ethics Committee (approval number: HE15-212). This muscle was present in the right medial arch of a 78-year-old white male cadaver. There was no equivalent muscle in the left foot. The foot did not have scars or pathology beyond age-related degeneration. No other variations were noted in the intrinsic or extrinsic musculature of either foot.

The anomalous muscle was formed from two slips (proximal and distal) on the plantar medial longitudinal arch, located deep to the AbH, and the tendons of the flexor digitorum longus and flexor hallucis longus. The tibialis anterior tendon inserted distal to the distal slip. Both slips passed superficially to the tibialis posterior tendon insertion and were proximal to the FHB origin.

The distal slip traveled transversely, while the proximal slip ran obliquely from the lateral attachment proximally to the medial attachment distally. The distal slip had muscular attachments to the short plantar ligament and some aponeurotic fibers to the long plantar ligament. The proximal slip had muscular attachments to the short plantar and spring ligaments, attaching near, yet remaining separate from, the tibialis posterior tendon, proximal tendinous origin of the lateral head of the FHB, and oblique head of the AdH. Proximal and distal slips combined to attach to the navicular tuberosity as aponeurotic fibers. Some extended to the tibionavicular portion of the deltoid ligament.

The medial plantar vessels and nerve passed from deep to superficial between the two slips (Fig. 1b). Small branches from these supplied the anomalous muscular slips.

## Discussion and conclusions

We present an anomalous muscle of the medial longitudinal arch that to our knowledge has not been previously reported. The position suggests a possible variation of an intrinsic muscle associated with the great toe or medial foot. Numerous variations in the abductor hallucis (AbH) [1, 4] and adductor hallucis (AdH) [2, 8] have been reported. For example, variant muscular slips of the AbH attaching to the proximal phalanges of the second and third toes [12], flexor hallucis longus tendon, or skin of the hallux [15] have been reported. Distally, AbH may also attach to the underlying tarsals, metatarsals, and fascial septae [6]. However, these are not discrete slips and are oriented with the main muscle belly rather than perpendicular to it as with the anomalous muscles described here.

Variable insertions of either head of the AdH into the first proximal phalanx have been described as opponens hallucis (OH) [12]. Macalister [15] regarded OH as frequently present in humans, either as a free slip or, more commonly, fused with the AdH. However, modern anatomical texts do not regard OH as a distinct muscle [16], nor do those variants resemble the OH of non-human primates [3, 5, 9]. However, all these muscle variants are described as running longitudinally, arising from the medial cuniform or navicular and inserting to the first metatarsal shaft or proximal hallucial phalanx base rather than running transversely.

The interpretation of the present anomalous muscle as an OH is also not clearly supported when considering development. The medial foot muscles develop from the superficial and deep layers of the plantar muscular blastema [7]. The superficial layer differentiates first, forming the AbH. The deep layer of the blastema splits to form two adjacent components—the medial and lateral heads of the FHB (initially formed as independent muscles)—and a component that becomes the variable first plantar interosseous muscle [7]. In comparison, the primordia for three consistent hand muscles are formed—the deep and superficial heads of the flexor pollicis brevis and the opponens pollicis. The latter lies lateral to the flexor pollicis brevis and deep to the abductor pollicis brevis. An equivalent OH does not occur in humans; the FHB and AdH variants proposed as such are more lateral and distal. The variable component of the deep plantar blastema may become continuous with the AdH oblique head or the first dorsal interosseous, or be considered the deep head of the FHB [7]. Čihák [7] termed this the musculis interosseous plantaris hallucis rather than accepting equivalence to the OH.

Direct comparison of development, anatomical variants, or function with non-human primates is difficult, as human bipedalism and loss of the hallucial grasp have resulted in musculoskeletal alterations in the foot [11]. However, this variant does not appear situated to produce opposition of the hallux and may act as a tensor of the medial longitudinal planar arch. The transverse orientation of the distal slip, with its attachments on the short and long plantar ligaments and navicular tuberosity, combined with the oblique orientation of the proximal slip, attaching to the proximal tendinous origin of the lateral head of the FHB, oblique head of AdH, and navicular tuberosity, suggests a relationship with the medial transverse tarsal joint and plantar calcaneonavicular (spring) ligament, potentially providing active support to stiffen the arch during locomotion. However, its small size belies meaningful movement at this area.

The medial plantar vessels and nerve pass from deep to superficial between the two slips (Fig. 1b), while giving off small branches to supply the muscular slips of this anomalous muscle. The medial plantar nerve arises under the flexor retinaculum, runs deep to the AbH, and, with the medial plantar vessels, travels between the AbH and flexor digitorum brevis [16]. Medial plantar nerve entrapment between the AbH and navicular, in the apex of the medial longitudinal arch, may result in medial plantar neuropraxia (Jogger’s foot) [10]. Passage of the medial plantar vessels and nerve between the two muscular slips here suggests a possible mechanism for nerve entrapment as these muscular slips contract.

Knowledge of variant foot muscles is important because of potential for pathology and for imaging interpretation. While analogous muscle slips described in non-human primates and the developmental origin of this anomalous muscle remain unclear, the location suggests a possible mechanism for medial nerve entrapment and function in supporting the medial longitudinal arch.

## Data Availability

Data sharing is not applicable to this article as no datasets were generated or analyzed during the current study.
